# Effective Treatment for Uncomplicated Urinary Tract Infections with Oral Fosfomycin, Single Center Four Year Retrospective Study

**DOI:** 10.3390/antibiotics9080511

**Published:** 2020-08-13

**Authors:** Miroslav Fajfr, Michal Balik, Eva Cermakova, Pavel Bostik

**Affiliations:** 1 Institute of Clinical Microbiology, University Hospital, Sokolska 581, 500 05 Hradec Kralove, Czech Republic; miroslav.fajfr@fnhk.cz; 2 Faculty of Medicine in Hradec Kralove, Charles University in Prague, Simkova 870, 500 38 Hradec Kralove, Czech Republic; michal.balik@fnhk.cz; 3 Department of Urology, University Hospital, Sokolska 581, 500 05 Hradec Kralove, Czech Republic; 4 Faculty of Medicine in Hradec Kralove, Department of Medical Biophysics, Charles University in Prague, Simkova 870, 500 38 Hradec Kralove, Czech Republic; cermakovae@lfhk.cuni.cz; 5 Faculty of Military Health Sciences, University of Defence, Trebesska 1575, 500 01 Hradec Kralove, Czech Republic

**Keywords:** fosfomycin, urinary infection, resistance

## Abstract

Fosfomycin represents a relatively old antibiotic, but it is experiencing a comeback in recent years. According to some studies, the increasing therapeutic use of this drug led to a rapid increase in the levels of resistance in bacteria causing urinary tract infection. In the presented study, levels of resistance to fosfomycin in more than 3500 bacterial isolates before and after fosfomycin introduction into therapeutic use in the Czech Republic and the clinical efficacy of treatment in 300 patients using this drug were assessed. The results show that the resistance levels to fosfomycin in *Escherichia coli* isolates before and after the drug registration were not significantly different (3.4% and 4.4%, respectively). In some other Gram-negative rods, such as otherwise susceptible *Enterobacter*, resistance to fosfomycin increased significantly from 45.6% to 76.6%. Fosfomycin treatment of urinary tract infections showed an excellent seven-day clinical efficacy (79.7%). However, when used to treat recurrent or complicated urinary tract infections, fosfomycin treatment was associated with high levels of infection relapse, leading to relapse in a total of 20.4% of patients during the first two months. This indicates that fosfomycin exhibits good efficacy only for the treatment of uncomplicated urinary tract infections

## 1. Introduction

Fosfomycin was introduced for the first time in 1969 as a product of *Streptomyces fradiae*, and it was also isolated from some members of the *Pseudomonas* species [1]. This drug was used for a long time in the treatment of urinary tract infections (UTIs), but the development of newer antibiotics led to a gradual decrease in its use. However, with the development of bacterial resistance to many antibiotics (i.e., β-lactam antibiotics, quinolones, aminglycosides) worldwide, fosfomycin is coming back as a viable alternative. Many countries adopted fosfomycin trometamol in their guidelines for urinary tract infection management not only for uncomplicated infections, but also for infections caused by multidrug-resistant bacteria [2,3]. However, this was associated, in turn, with a rapid increase in the resistance level of bacteria to fosfomycin, according to data from several countries. Data from Spain show an increase in Fosfomycin-resistant *Escherichia coli* extended-spectrum beta-lactamase (ESBL)-producing isolates from 4.4% in 2005 to 11.4% in 2009. However, the overall resistance level of all *Escherichia coli* isolates (ESBL producers and ESBL non-producers) to fosfomycin in this study remained low at 2.9% in 2019. Another study from China showed that a high percentage of carbapenemase (KPC)-producing *Klebsiella pneumoniae* isolates harbor FosA3 (34%)*,* which would suggest the loss of effectiveness of fosfomycin and the multidrug resistance (MDR) of these bacteria. Data from Poland show the overall susceptibility of *E. coli* isolates to fosfomycin to be 62.2% in complicated UTIs and 77.6% in uncomplicated UTIs [4,5,6]. These data, thus, indicate a potential problem in the use of fosfomycin in the treatment of nosocomial acquired urinary tract infections (UTIs).

In the Czech Republic, fosfomycin trometamol was not licensed for clinical use until October 2014. The resistance of bacteria was, therefore, very low, as described in our previous work and as also reported from other countries [7,8]. In 2015, oral fosfomycin trometamol was implemented into the Czech national UTI treatment guidelines as a drug of second choice for uncomplicated infections of the lower urinary tract. Here, we describe a study of the clinical effects of fosfomycin trometamol use in the University Hospital in Hradec Kralove, Czech Republic, and we evaluate trends in bacterial susceptibility to fosfomycin during the first four years of its use for the treatment of UTIs.

## 2. Results

### 2.1. Susceptibility of Bacteria Causing UTI to Fosfomycin

**Figure 1:**
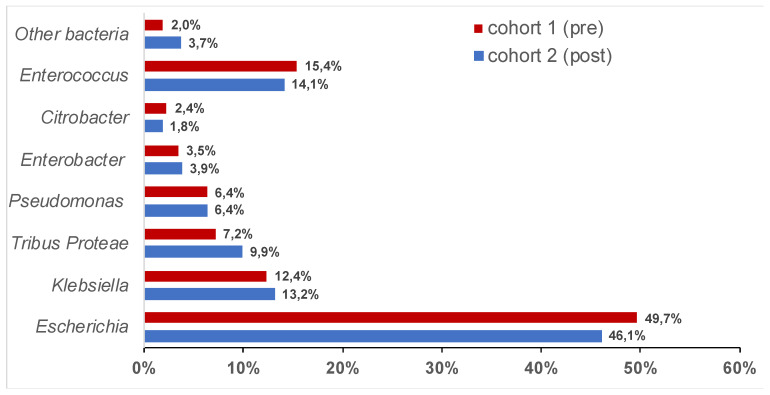
Proportional representation of bacteria isolated from urine samples in Cohort 1 (before fosfomycin registration) and Cohort 2 (after fosfomycin registration). Bacteria were stratified by families; Tribus *Proteae* includes *Proteus* spp., *Morganella* spp., and *Providentia* spp.

The prevalence of individual bacterial strains isolated from urine samples was similar when comparing samples from two patient cohorts before (Cohort 1) and after (Cohort 2) the introduction of fosfomycin into the treatment of UTIs. The most frequent bacterium isolated was *Escherichia coli*, with levels of 46.1% and 49.7%, respectively. Other bacteria isolated more frequently than in 10% of samples were *Enterococcus faecalis* and *Klebsiella* species. The similar bacterial stratification allowed for a comparison of both cohorts in the next step. The entire spectrum of bacteria isolated is shown in Figure 1.

The general level of resistance (beta-lactamase production, resistance to fluoroquinolones, and multidrug resistance) of Gram-negative bacteria showed a notable increase over time when analyzed in the older (Cohort1) and more recent (Cohort 2) samples (Table 1). Thus, the number of high-risk beta-lactamase-positive (ESBL, AmpC, and K1) bacterial strains rose from 11.7% to 19.8%, and the number of fluoroquinolone-resistant ones rose from 25.9% to 33.8%. *Escherichia* species isolates generally showed lower levels of resistance to antibiotics with no significant increase over time (*p* = 0.23304). The highest level of resistance was detected in *Klebsiella* species isolates, but there was also no significant increase when comparing the two cohorts (*p* = 0.53870). On the contrary, multidrug-resistant (MDR) *Pseudomonas* isolates (defined as resistant to beta-lactams, carbapenems, and fluoroquinolones) showed a markable and statistically significant increase over time from 5.6% to 13.3% (*p* = 0.00026).

The analysis of resistance of the three most common Gram-negative rods to fosfomycin and its development over time is shown in Table 2. Eesistance to fosfomycin was generally low in all *Escherichia* isolates, with no statistically significant differences before and after the fosfomycin registration, with levels of 4.4% and 3.4%, respectively. However, both *Klebsiella* and *Enterobacter* isolates showed statistically significant increases in resistance after the introduction of fosfomycin. Thus, in the high-risk beta-lactamase-producing *Klebsiella* isolates, the resistance to fosfomycin increased from 87% to 95.2% (*p* = 0.0467). In the *Enterobacter* isolates with no other resistance detected, fosfomycin resistance increased dramatically from 45.6% to 76.6% (*p* = 0.0105). However, the overall increase in fosfomycin resistance in all *Enterobacter* isolates was also high and statistically significant (55.2% vs. 74.4%; *p* = 0.0468). Taken together, of the more prevalent bacterial isolates from the UTIs that showed other drug resistances (high-risk beta-lactamases production or resistance to fluroquinolones), only the *E. coli* isolates were sufficiently susceptible to fosfomycin both before and after its introduction into the treatment regimes.

### 2.2. Clinical Efficacy of Fosfomycin

The clinical effects were analyzed in 300 patients treated with fosfomycin trometamol for UTIs (Cohort 3). The basic demographic data of the patients are shown in Table 3. In total, 428 fosfomycin trometamol doses were prescribed, i.e., 1.43 doses on average for a patient, with a maximum of four doses per patient. Altogether, 66.0% (*n* = 198) of patients were administered a single dose and 34.0% (*n* = 102) of patients received multiple doses. Multiple-dose therapy was used mostly in patients with complicated (in 51.0% of these cases) or recurrent (in 43.7% of these cases) infections, but it was relatively rare in patients with uncomplicated infections (in 19.5% of these cases). In 50.0% of patients, bacterial cultivation was performed before fosfomycin treatment, while 50.0% of patients were treated empirically without the culture. The type of UTI was clearly specified in 234 cases. In 67.1% (*n* = 157) of patients, complicated/recurrent UTIs were diagnosed, while 32.9% of patients had an uncomplicated UTI (all females). Nearly 30% of patients (*n* = 88) did not come for a follow-up visit, and the therapeutic effect could not be evaluated.

The clinical effects of fosfomycin treatment in the individual groups are shown in Table 4. Among those, where the effects could be evaluated, fosfomycin had an immediate curative effect in 79.7% of cases (*n* = 169); however, in 20.1% of cases (*n* = 43), the drug had no positive effect. The highest fosfomycin immediate cure rate was observed in patients with infections caused by *Escherichia* species (69.8%) and *Enterococcus faecalis* (50.0%). In cases with no detectable antibiotic effect of fosfomycin, the bacterial culture (before antibiotic administration or control culture) was performed in 79.1% of patients. In one-third of these patients, susceptible bacteria were detected (*Escherichia* species or *Enterococcus faecalis*), while the culture was negative in 26.5% of cases (*n* = 9) and 14.7% (*n* = 5) had mixed culture; the remaining cases involved bacteria with no EUCAST susceptibility breakpoints of fosfomycin in the disc diffusion test (*Klebsiella pneumoniae*, *Pseudomonas aeruginosa*, *Streptococcus agalactiae*).

The second evaluation criterion was the recurrence level after the administration of fosfomycin up to one year after drug use. Sufficient data could only be obtained from 186 patients, since 114 patients escaped the evidence of the Urology Clinic. The fact, the relatively high number of patients with uncomplicated UTIs that were not present for their follow-up visit may indicate that the antibiotic had a curable effect (according to standard procedures, in the case of successful treatment, no follow-up visit is needed). However, since there was no evidence for it, these patients were excluded from the analysis. From the included cases, 40.9% (*n* = 76) had no relapse of the same UTI, 21.5% (*n* = 43) had a relapse during the first two months after fosfomycin treatment, and 14.5% (*n* = 27) of patients had recurrence in the time period of 2–12 months after fosfomycin administration. From the patients with an early relapse, 57.5% were administered a single dose and 42.5% were administered multiple doses of the drug. In most cases, fosfomycin had no effect or the UTI showed early recurrence in patients with complicated UTIs (complicated UTIs created more than 90% of acute fosfomycin failure cases or cases with early recurrence). However, in patients with uncomplicated UTIs, cure failure or recurrence was very rare (5.6–11.1%). Thus, the statistical evaluation showed a much lower relapse rate in the group with uncomplicated UTIs in comparison to complicated or recurring UTIs for all three analyzed time periods, which was highly significant (*p* = 0.00004).

The dosage impact on immediate effect was not seen, as the same percentage of single-dose therapy (60.4% and 60.3%) was found in cases with no cure effect and with immediate cure effect.

## 3. Discussion

In this study, urine samples from patients experiencing urinary tract infections were analyzed with the aim to assess the efficacy of fosfomycin treatment and to identify whether the introduction of this drug into the treatment regime led to any increase in antimicrobial resistance to fosfomycin. Although many patients did not come for their follow-up visits, thereby decreasing the numbers available for analysis, the size of our cohort is comparable to the studies cited throughout this report and elsewhere [9]; therefore it represents, in our opinion, a valid set of data, which can be used for further treatment guidance.

Our study showed a relatively stable proportion of bacterial species isolated from the UTIs with a dominance of *E. coli*, *Enterococcus faecalis*, and *Klebsiella* species. These bacterial species represent the most prevalent isolates from urinary tract infections worldwide [10,11]. Previous studies showed that fosfomycin remains very effective for the treatment of urinary tract infections caused by various bacteria, including *E. coli* or enterococci, in which the susceptibility to the drug was reported around 90% [7,10,12,13]. This was fully confirmed by our data, where the overall susceptibility in both cohorts (pre- and post-fosfomycin introduction) was 96.5% for all *E. coli* isolates and 100.0% for *Enterococcus faecalis* (data not shown). While there is some evidence of similarly high susceptibility to fosfomycin in ESBL-producing bacteria [13], most studies showed significantly lower numbers (around 80%) than in non-beta-lactamase producers [7,10,14]. This overall trend was also reflected in the data presented here. For example, while the resistance level in non-ESBL isolates of *E. coli* was 3.0%, the resistance increased to 9.7% in ESBL-positive isolates.

According to a previously published meta-analysis, the risk of selecting resistant mutants during fosfomycin monotherapy was calculated at 3.4% [15]. This is likely why some countries, such as China, Spain, Turkey, and some regions of India, reported alarmingly increasing fosfomycin resistance levels associated with its increased application. Thus, the fosfomycin resistance levels of non-beta lactamase-producing *E. coli* reached as high as 15% in some of these countries [16]. On the contrary, in some other areas, including Japan and the majority of the European and American countries, fosfomycin resistance was maintained at low levels around 4.5% despite the increase in drug use [12,16,17]. Moreover, reports from Hungary showed inconsistent fosfomycin resistance levels of *Citrobacter*, *Enterobacter*, and *Serratia* isolates over five years of study, with an overall resistance level of 9.3% in outpatient and 13.8% in inpatient settings. The resistance level of Tribus *Proteae* members to fosfomycin from the same country was calculated to be 18.7% in inpatients and 30.3% in outpatients over 10 years of study [18,19]. A study from Israel conducted during a time period almost identical to that in our study showed a notable increase in overall fosfomycin resistance in Gram-negative bacteria from 20.7% in 2015 to 30.9% in 2016 [20]. Our data show low resistance levels in *Escherichia coli* isolates and high resistance levels in isolates of *Klebsiella* and *Enterobacter.* With the exception of *Enterobacter,* the resistance levels were, however, maintained over time with no demonstrated effect of increased fosfomycin use after its registration. The reason for this relatively stable susceptibility to fosfomycin in our country can be explained by the relatively infrequent prescription of this drug in the Czech Republic overall. In our system, fosfomycin is fully paid for by patients with no contribution from the health insurance system; as such, it is not usually the first drug of choice.

Fosfomycin was reported as a relatively effective drug for the treatment of UTIs. For example, one United States (US) study reported a microbiological cure rate of 59.0% [21] and a similar Chinese study showed an overall 15-day cure rate of 65.07%, with variability in accordance with gender, age, and infection type [22]. Our data show an overall seven-day clinical cure rate of 79.7%, which is slightly higher than the rates mentioned above. One potential explanation for the better results in our study may lie in the differences in the spectrum of bacteria isolated. Thus, in our cohort extended-spectrum beta-lactamase-producing bacteria represented only 14.0% of isolates, while, in the studies above, the numbers of multidrug-resistant isolates were higher. Our data also confirmed the correlation between the treatment efficacy and the type of UTI. Thus, a report from China showed the effectivity of fosfomycin treatment in 97.71% of uncomplicated and 62.69% of complicated UTIs [22], while our data show efficacies of 94.8% and 70.2%, respectively. A high percentage of our patients showed relapses of UTIs in our study. In total, 21.5% of patients treated with fosfomycin experienced a relapse of infection in the first two months after therapy. Fosfomycin therapy in the complicated UTIs was, according to our data, ineffective in 51.3% of cases (i.e., the drug has no effect, or there is early relapse of symptoms within two months). In the treatment of complicated UTIs or UTIs caused by multidrug-resistant microbes, many studies suggested multiple dose regimens of fosfomycin [1,21,22,23,24,25,26] with three-dose regimens being the most common. Patients in our cohorts were mostly treated with a single-dose regimen; however, about one-third of them received multiple doses. Our data support the approach to apply multiple doses of fosfomycin in complicated UTIs, because early relapses were much more frequent when single-dose regimens were used. The treatment assessment studies mostly showed multiple dose regimens within the range of 1.4 to 2.0 doses per patient in accordance with the NICE guidelines, with administration of single-dose treatments in female and double-dose regimens in male patients. In our study, the average regimen consisted of 1.4 doses per patient, and multiple-dose regimens were used more frequently in the male subgroup (in 48.7% of cases, compared to 29.0% in females), which is fully in accordance with other published data [9,21,27]. Some studies showed a significantly higher failure level in UTIs associated with *Klebsiella* infections than in those caused by *E. coli* [9,27]. Similarly, we found a higher failure rate in infections caused by *Klebsiella* isolates (35.7%) than *E. coli* (17.0%), but the highest treatment failure rate of 44.4% was present in UTIs associated with bacterial mixtures (combinations of *Kl. pneumoniae*, *E. coli*, *Proteus* spp., or *Enterococcus*).

The strength of the presented study is in the comparison of resistance levels in a naïve bacterial population (before oral fosfomycin use) with those in a bacterial population after the introduction and wide use of fosfomycin in treatment regimens. The other important aspect of the presented data lies in the evaluation of oral fosfomycin cure effectivity in urinary tract infections. Although our study had a limitation in the relatively low number of patients (in part due to the fact that many patients did not come for their follow-up visits), the size of our cohort is comparable to those cited in studies throughout this report and elsewhere (e.g., 24). The fact, that a relatively high number of patients with uncomplicated UTIs did not present for their follow-up visit may indicate that the antibiotic had a curable effect. According to standard procedures, in the case of a successful treatment, no follow-up visit is required; thus, the long-term curable effect of oral fosfomycin in uncomplicated urinary tract infections may, in fact, be even better. Nevertheless, since this could not be supported by any evidence, these patients were excluded from the analysis.

## 4. Materials and Methods

### 4.1. Patient Cohorts and Samples

Urine samples were obtained from patients treated at the Urology Clinic, University Hospital in Hradec Kralove, Czech Republic during the period 2013–2018. Samples were collected from patients with UTI signs including dysuria, flank pain, urinary frequency or urgency, leukocyturia and/or positive culture, and fever. Negative samples and all samples positive for the following microorganisms were excluded from the study: yeast, coagulase-negative staphylococci (with the exception of *Staphylococcus saprophyticus*), and other non-pathogenic bacteria (e.g., lactobacilli, corynebacteria). Moreover, all positive isolates without available fosfomycin susceptibility data were excluded.

The antimicrobial resistance of bacteria to fosfomycin prior to and following the drug’s introduction into the UTI treatment guidelines was evaluated in samples from two cohorts of patients. Cohort 1 (before fosfomycin registration) consisted of samples collected in the period 2013–2014 and contained a total of 594 bacterial isolates. Cohort 2 (post fosfomycin introduction) consisted of 2935 bacterial isolates obtained from patients treated at the Urology Clinic in the years 2015–2018. The evaluation of fosfomycin treatment efficacy was performed in 300 patients (Cohort 3) treated at the Urology Clinic in the period 2015–2018 for UTIs. Of these patients, 25.7% (*n* = 77) had uncomplicated UTIs (defined as acute cystitis in women or acute cystitis in young men), 22.0% had undefined UTIs, and 52.3% (*n* = 157) had complicated UTIs (defined as recurrent UTIs, UTIs after urinary tract surgery, UTIs in men with benign prostate hyperplasia or carcinoma, or UTIs associated with lithiasis). Data from the electronic records of patients and microbiological test results were used for the assessment of clinical outcome. The patient data were strictly anonymized; thus, according to the Ethics Committee of the University Hospital, no patient consent was needed.

### 4.2. Susceptibility Evaluation

Standard Mueller–Hinton agar (Trios, Czech Republic) and a disc diffusion test with a 200 µg fosfomycin disc with the 50 µg of G-6-P (glucose-6-phosphate) supplement were used according to the EUCAST and CLSI guidelines. For the susceptibility evaluation of *Enterobacteriales*, the EUCAST guideline was applied (EUCAST Clinical breakpoint tables v. 09, valid from 2019-01-01). *Escherichia coli* isolates were considered susceptible to fosfomycin in the case of zone diameters ≥ 24 mm. Similar criteria were adopted to show the resistance levels in two other Gram-negative bacteria (*Klebsiella* species and *Enterobacter* species) for illustration, as used in some previous publications [10,14]. For other bacteria (*E. faecalis*), the CLSI susceptibility criteria (M100 S29, valid from 2019-01-01) were used. The following susceptibility criteria for *E. faecalis* were used: zone diameters ≥16 mm indicated susceptible isolates and zone diameters ≤ 12 mm indicated resistant ones.

### 4.3. Clinical Efficacy Evaluation

The clinical effects of the applied treatment were evaluated by urologists according to the criteria described below. The classification of UTIs was adopted from EAU Urological Infection Guidelines 2020 with three groups presented: uncomplicated, complicated, and recurrent (classification available on EAU website: https://uroweb.org/guideline/urological-infections/#3). The first assessment was the drug’s acute effect, meaning the curative effect in the first seven days after drug administration; cases were classified as cured (the symptoms of UTI disappeared and/or negative culture result in control sample), no effect or acute failure (without any positive effect; the UTI symptoms were present despite the treatment), or not assessed (no data available, patients did not come for follow-up). The second criterion was the recurrence of the same clinical unit in the first year after drug administration with a focus on the first two months, which would indicate a relapse and treatment failure. All these data were correlated with the culture results, dose regime, and type of UTI.

### 4.4. Statistics

Statistical evaluation was performed using Pearson’s chi-squared test with a significance level of *p* = 0.05. In the case of low numbers for the chi-squared test, the modified Fisher’s exact test was used. The statistical evaluations were performed using the NCSS 11 Statistical Software (2016) (NCSS, LLC. Kaysville, UT, USA, ncss.com/software/ncss).

## 5. Conclusions

Our data confirmed the high susceptibility of Escherichia coli and Enterococcus faecalis to fosfomycin. The results did not show any increase of resistance levels after the registration and introduction of this drug to UTIs treatment regimes. The oral fosfomycin showed a high treatment efficacy in uncomplicated urinary tract infections. However, in complicated or recurrent infections the treatment led to a relatively high recurrence index.

## Figures and Tables

**Table 1:** Resistance levels of the most frequent pathogenic bacteria from urine samples before (Cohort 1) and after (Cohort 2) fosfomycin registration. Brackets indicate statistically significant differences (*p* < 0.05) between Cohort 1 and Cohort 2.

Bacteria	Cohort 1										Cohort 2									
	BL (ESBL, AmpC, K1)		iAmpC		FQR		MDR		NO RES		BL (ESBL, AmpC, K1)		iAmpC		FQR		MDR		NO RES	
	%	No.	%	No.	%	No.	%	No.	%	No.	%	No.	%	No.	%	No.	%	No.	%	No.
*Citrobacter* spp.	12.1	4	(0.0)	0	9.1	3	0.0	0	(78.8)	26	10.5	10	(25.3)	24	12.6	12	0.0	0	(51.6)	49
*Enterobacter* spp.	19.2	10	(0.0)	0	19.2	10	0.0	0	(61.6)	32	26.9	56	(24.5)	51	14.9	31	0.0	0	(33.7)	70
*Escherichia coli*	5.4	37	0.0	0	17.0	116	0.0	0	77.6	530	7.3	166	0.2	4	17.2	392	0.0	0	75.3	1714
*Klebsiella* spp.	29.0	64	0.0	0	37.5	83	0.0	0	33.5	74	30.8	252	0.4	3	33.2	272	0.0	0	35.6	291
*Pseudomonas* spp.	0.0	0	0.0	0	(42.7)	38	(5.6)	5	51.7	46	0.0	0	0.0	0	(21.9)	66	(13.3)	40	64.8	195
Tribus *Proteae*	2.1	2	(0.0)	0	27.8	27	0.0	0	(70.1)	68	4.3	21	(11.6)	57	32.4	159	0.0	0	(51.7)	254

BL—high-risk beta-lactamases (ESBL, AmpC, K1); iAmpC—inducibile AmpC beta-lactamases; FQR—fluoroquinolone resistance; MDR—multidrug resistance; NO RES—no resistance; Tribus *Proteae*—*Proteus* spp., *Morganella* spp., and *Providentia* spp.

**Table 2:** Resistance levels of the most frequent Gram-negative bacteria from urine samples to fosfomycin before (Cohort 1) and after (Cohort 2) fosfomycin registration. Brackets indicate statistically significant differences (*p* < 0.05) between Cohort 1 and Cohort 2.

Bacteria		Cohort 1		Cohort 2	
	Resistance	Fosfomycin Resistant (%)	Total	Fosfomycin Resistant (%)	Total
*Escherichia coli*	BL	16.0	25	8.7	161
	FQR	0.0	43	3.8	262
	NO	4.0	273	2.8	1702
*Escherichia* coli in total	ALL	4.4		3.4	
*Klebsiella* species	BL	(87.0)	54	(95.2)	165
	FQR	83.3	12	90.6	32
	NO	74.5	47	79.6	147
*Klebsiella* species in total	ALL	81.4		88.1	
*Enterobacter* species	BL	85.7	7	81.0	21
	FQR	0.0	0	100.0	1
	iAmpC	0.0	0	53.8	13
	NO	(45.6)	22	(76.6)	47
*Enterobacter* species in total	ALL	(55.2)		(74.4)	

BL—high-risk beta-lactamases (ESBL, AmpC, K1); iAmpC—inducible AmpC beta-lactamases; FQR—fluoroquinolone resistance; NO—no resistance.

**Table 3:** Basic demographic data of patients from Cohort 3 (patients evaluated for fosfomycin treatment efficacy). UTI—urinary tract infection.

Demographic Characteristics of Patients			
Variables	Parameters		%
Gender	Male	77	25.7
	Female	223	74.3
Age (years)	Average	51 years	Range 16–93 years
UTI type *	Uncomplicated	77	32.9
	Recurrent	110	47.0
	Complicated	47	20.1

* Only patients with available data.

**Table 4:** Results of clinical efficacy evaluation of fosfomycin treatment, case distribution according to UTI type, and clinical follow-up after fosfomycin administration. Statistically significant differences (at *p* < 0.05) are shown in brackets.

	Relapse (in %)				Total
UTI Type	No Relapse/Fully Cured	No Effect	Relapse ≤2 M	Relapse 2–12 M	
**Complicated**	38.1	33.3	16.7	11.9	42
**Recurrent**	29.6	23.2	28.7	18.5	108
**Uncomplicated**	(77.8)	11.1	(5.6)	5.6	36

M—months.
